# Neuroprotective and Anti-Apoptotic Effects of CSP-1103 in Primary Cortical Neurons Exposed to Oxygen and Glucose Deprivation

**DOI:** 10.3390/ijms18010184

**Published:** 2017-01-18

**Authors:** Vanessa Porrini, Ilenia Sarnico, Marina Benarese, Caterina Branca, Mariana Mota, Annamaria Lanzillotta, Arianna Bellucci, Edoardo Parrella, Lara Faggi, Pierfranco Spano, Bruno Pietro Imbimbo, Marina Pizzi

**Affiliations:** 1 Department of Molecular and Translational Medicine, University of Brescia, Viale Europa 11, 25123 Brescia, Italy; ilesar@libero.it (I.S.); marina.benarese@unibs.it (M.B.); c.branca@unibs.it (C.B.); m.coelhodamota@unibs.it (M.M.); annamaria.lanzillotta@unibs.it (A.L.); arianna.bellucci@unibs.it (A.B.); edoardo.parrella@unibs.it (E.P.); lara.faggi@unibs.it (L.F.); pierfranco.spano@unibs.it (P.S.); 2 Department of Neurorehabilitation, IRCCS San Camillo, Via Alberoni 70, 30126 Venice, Italy; 3 Research & Development, Chiesi Farmaceutici, Via Palermo 26/A, 43100 Parma, Italy; b.imbimbo@chiesi.com

**Keywords:** CSP-1103, CHF5074, oxygen-glucose deprivation, ischemia, caspase-3, nuclear factor κB (NF-κB), p38, amyloid precursor protein intracellular domain (AICD)

## Abstract

CSP-1103 (formerly CHF5074) has been shown to reverse memory impairment and reduce amyloid plaque as well as inflammatory microglia activation in preclinical models of Alzheimer’s disease. Moreover, it was found to improve cognition and reduce brain inflammation in patients with mild cognitive impairment. Recent evidence suggests that CSP-1103 acts through a single molecular target, the amyloid precursor protein intracellular domain (AICD), a transcriptional regulator implicated in inflammation and apoptosis. We here tested the possible anti-apoptotic and neuroprotective activity of CSP-1103 in a cell-based model of post-ischemic injury, wherein the primary mouse cortical neurons were exposed to oxygen-glucose deprivation (OGD). When added after OGD, CSP-1103 prevented the apoptosis cascade by reducing cytochrome c release and caspase-3 activation and the secondary necrosis. Additionally, CSP-1103 limited earlier activation of p38 and nuclear factor κB (NF-κB) pathways. These results demonstrate that CSP-1103 is neuroprotective in a model of post-ischemic brain injury and provide further mechanistic insights as regards its ability to reduce apoptosis and potential production of pro-inflammatory cytokines. In conclusion, these findings suggest a potential use of CSP-1103 for the treatment of brain ischemia.

## 1. Introduction

Stroke is one of the major causes of mortality and disability worldwide [1]. Presently, there are no clinically effective therapies for stroke recovery, and current treatments offer only limited benefits, making ever more urgent the need to develop new therapies. Apoptosis and necrosis are key mechanisms that lead to cell death after cerebral ischemia. In the ischemic core in particular, most of the cells die of necrosis, while apoptosis is mainly involved with the penumbra, the border of the ischemic area, where the levels of energy and oxygen are sufficient to support apoptotic processes [2,3,4]. In fact, signs of apoptosis exist, including cytochrome c release, activation of caspases-3 and -9, and terminal deoxynucleotidyl transferase dUTP nick end labeling (TUNEL) positivity, thus making this cascade a potential target for neuroprotection [5,6,7,8].

CSP-1103 (1-(3′,4′-dichloro-2-fluoro (1,1′-biphenyl)-4-yl)-cyclopropanecarboxylic acid) is an orally bioavailable, brain penetrating, non-steroidal anti-inflammatory drug (NSAID) derivative with markedly reduced cyclooxygenase (COX) inhibitory activity [9], in contrast to classic NSAIDs such as ibuprofen, in development for the treatment of Alzheimer’s disease (AD) and other neurodegenerative disorders by CereSpir Incorporated.

CSP-1103 was originally found to limit the β-amyloid (Aβ) plaque burden and ameliorate cognitive deficits when chronically administered to transgenic mouse models of AD [9,10,11,12]. Based on the results of preclinical and clinical investigations [13,14,15], CSP-1103 has been hypothesized to reduce the production and release of pro-inflammatory cytokines by microglia and to enhance microglial phagocytic capacity via an effect on astrocyte-microglia cross-talk. Specifically, CSP-1103 is thought to reduce the availability of the astrocytic-signaling molecule soluble CD40 ligand (sCD40L), which normally binds the microglial receptor CD40 on the cell surface. The CD40 ligation is a recognized mechanism driving the pro-inflammatory phenotype of microglia, including the release of cytokines such as tumor necrosis factor α (TNF-α), triggering the free radical-mediated tissue damage and limiting the microglial phagocytic activity [16]. Preclinical evidence shows that CSP-1103 reduces the levels of a number of pro-inflammatory microglial markers, including inducible nitric oxide synthase (iNOS), interleukin 1 β (IL-1β), and TNF-α, while stimulating the transcription of proteins involved in phagocytosis, such as triggering receptor on myeloid cells 2 (TREM2) [14]. A Phase 1 2-week study in healthy volunteers and a Phase 2a 12-week study in patients with mild cognitive impairment confirmed in humans the TNF-α inhibiting properties of CSP-1103 and showed a corresponding effect on sCD40L levels in the cerebrospinal fluid (CSF) [13,15]. Lower CSF total tau levels were also noted [17], while no effects were seen on soluble Aβ species (Aβ40 and Aβ42) [13,15].

The observation that CSP-1103 binds the amyloid precursor protein intracellular domain (AICD) with sub-micromolar affinity and inhibits AICD nuclear translocation and interaction with target gene promoters [18], provides a plausible mechanism to explain the various beneficial effects of the compound through a single molecular target. AICD is a transcriptional regulator that affects the production of proteins involved in inflammation, intracellular trafficking, and apoptosis [19]. Among the AICD target genes, CSP-1103 was shown to impair the expression of pro-apoptotic tetraspanin *KAI1*/*CD82* in Tg2576 mice [18]. Moreover, “pretreatment” with CSP-1103 was found to reduce apoptosis in diverse experimental settings, including in the SH-SY5Y neuroblastoma cells exposed to Aβ25-35 or Tumor Necrosis Factor Related Apoptosis Inducing Ligand (TRAIL) [20] or the primary hippocampal neurons exposed to oxygen glucose deprivation (OGD) [21].

These findings prompted us to expand on the anti-apoptotic effects of CSP-1103 by investigating its activity in a diverse experimental settings mimicking the therapeutic scheme for the “post-ischemic” injury. By using primary cortical neurons exposed to OGD as a cell-based model of cortical brain ischemia, we tested the neuroprotective and anti-apoptotic effects of CSP-1103, when added in the post-OGD period, and the signaling cascade involved. The activity of CSP-1103 was compared to that of ibuprofen, a classical NSAID with potent COX inhibitory activity.

## 2. Results

### 2.1. CSP-1103, but Not Ibuprofen, Reduces Necrosis Induced by OGD in Primary Cortical Neurons

**Figure 1:**
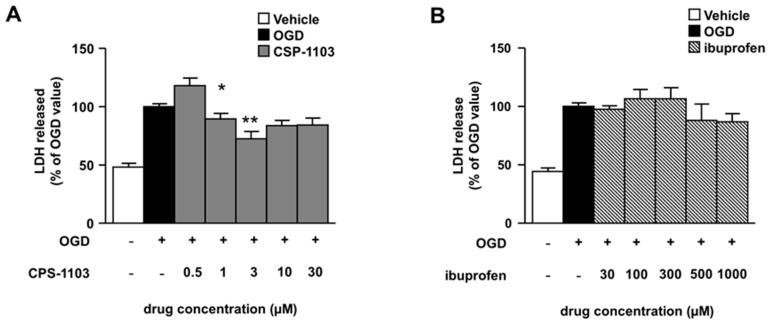
Neuroprotective effect of CSP-1103, but not ibuprofen, in cortical neurons exposed to oxygen glucose deprivation (OGD). (**A**) Cortical neurons were exposed to 3 h OGD and neuronal death was assessed after 24 h recovery by the lactate dehydrogenase (LDH) assay. CSP-1103, added after the OGD period, increased the survival of injured neurons at different concentrations (1 and 3 μM); (**B**) Lack of neuroprotective effect of ibuprofen in cortical neurons exposed to 3 h OGD. Different doses of ibuprofen were added after OGD and the neuronal death was measured by the LDH assay 24 h later. Values are expressed as percentage of the LDH released by the cells exposed to OGD. * *p* < 0.05, ** *p* < 0.01 vs. OGD value. +, presence of OGD; −, absence of OGD or treatment.

Following OGD, primary cultures of mouse cortical neurons have been shown to undergo apoptosis prior to necrosis. Neuronal cells display TUNEL-positivity and release of cytochrome c in the cytosol within 6 h after the OGD, in the absence of lactate dehydrogenase (LDH) release. A secondary necrosis was revealed by the progressive release of cellular LDH that became clearly detectable in the culture medium 24 h after the OGD [22,23]. The effects of CSP-1103 and ibuprofen on neuronal injury were firstly evaluated at the end of the 24 h recovery period by measuring LDH release (Figure 1).

Cortical neurons were exposed to CSP-1103 or ibuprofen in the post-OGD period, a condition which mimics in vitro the therapeutic scheme for in vivo brain ischemia. CSP-1103, tested at concentrations ranging from 0.5 μM to 30 μM, displayed maximal neuroprotection at 3 μM (Figure 1A). Conversely, no protection was elicited by ibuprofen at any concentration tested in the range 30–1000 μM (Figure 1B). No toxic effects were elicited by CSP-1103 and ibuprofen when added to naïve neuronal cells at the same concentration range (Figure S1).

### 2.2. CSP-1103, but Not Ibuprofen, Prevents Caspase-3 Activation Induced by OGD in Primary Cortical Neurons

**Figure 2:**
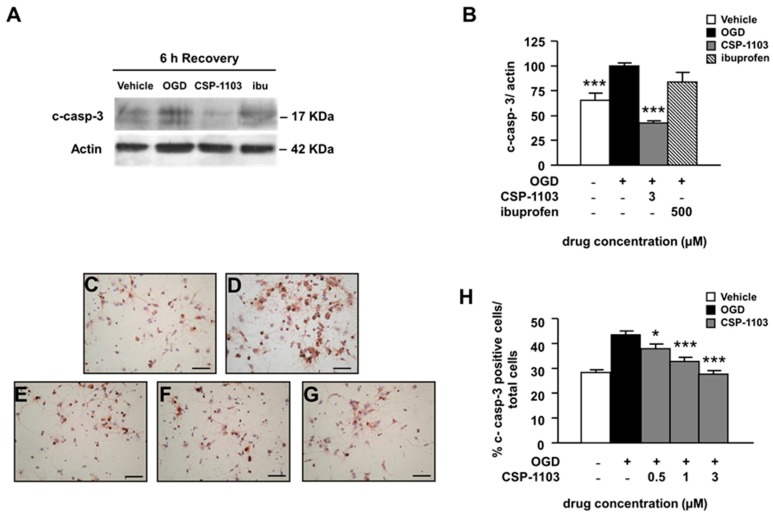
Effect of CSP-1103 and ibuprofen on caspase-3 cleavage in cortical neurons exposed to OGD. (**A**) Representative images and (**B**) densitometry analysis of western blot (WB) for c-casp-3 in cytosolic extracts of cells exposed to OGD with or without drugs during 6 h recovery. CSP-1103, but not ibuprofen, was able to reduce c-casp-3 to the basal level. Bars (mean ± SEM) represent the percentage of the casp-3/actin ratio, relative to the OGD value. (**C**–**G**) Representative images of immunocytochemistry for c-casp-3 and (**H**) percentages of c-casp-3-positive cells counted after 24 h recovery ((**C**) vehicle; (**D**) OGD; (**E**) CSP-1103 0.5 μM; (**F**) CSP-1103 1 μM; and (**G**) CSP-1103 3 μM). CSP-1103 reduced the number of c-casp-3 immunopositive cells. Bars (mean ± SEM) represent the percentage of c-casp3-positive neurons compared to the total cell number. * *p* < 0.05, *** *p* < 0.001 vs. OGD value. +, presence of OGD; −, absence of OGD or treatment.

To examine the apoptotic cascade induced by OGD, we measured the activity of caspase-3, a member of the cysteine-dependent, aspartate-specific, proteolytic enzymes family that plays a central role in the propagation of the apoptotic processes. We checked the level of cleaved caspase-3 protein (c-casp-3) in cytosolic extracts of neurons exposed to OGD and treated with the vehicle or the drugs for 6 h in the post-OGD period. Data analysis from western blot (WB) revealed that CSP-1103 at 3 µM significantly reduced caspase-3 cleavage (Figure 2A,B). Conversely, ibuprofen did not modify caspase-3 cleavage, in line with the lack of neuroprotective activity of the drug shown by the LDH release assay (Figure 2A,B).

Furthermore, we checked the immunoreactivity to c-casp-3 in neurons exposed to CSP-1103 (0.5–3 µM) for 24 h in the post-OGD period. Cells were processed for immunocytochemistry, using an antibody specific for c-casp-3 and counterstained with hematoxylin. The data showed increased immunoreactivity for c-casp-3 in cells exposed to OGD. The immunoreactivity was reduced in neurons treated with CSP-1103 at concentrations ≥0.5 μM (Figure 2C–H).

### 2.3. CSP-1103, but Not Ibuprofen, Prevents Cytochrome C Release Induced by OGD in Primary Cortical Neurons

**Figure 3:**
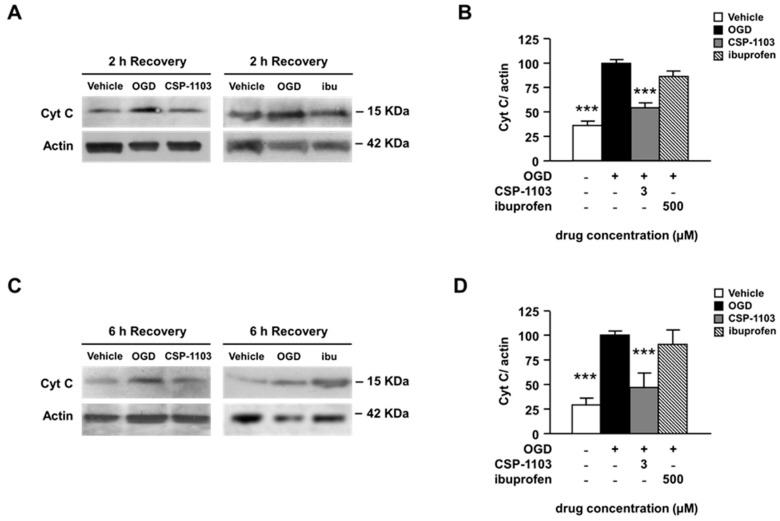
Effect of CSP-1103 and ibuprofen on cytochrome c in cortical neurons exposed to OGD. (**A**) Representative images and (**B**) densitometry analysis of WB for Cyt C in cytosolic extracts after 2 h of recovery. CSP-1103, but not ibuprofen, reversed the OGD-induced cytochrome c release from mitochondria after 2 h of recovery. Bars (mean ± SEM) represent the percentage of the Cyt C/actin ratio relative to the OGD value. (**C**) Representative images and (**D**) densitometry analysis of WB for Cyt C in cytosolic extracts after 6 h of recovery. The pro-apoptotic release of cytochrome c was blocked by CSP-1103 treatment of neurons after 6 h of recovery. Bars (mean ± SEM) represent the percentage of the Cyt C/actin ratio, relative to the OGD value. *** *p* < 0.001 vs. OGD value. +, presence of OGD; −, absence of OGD or treatment.

As a further marker of apoptosis, we investigated the levels of cytochrome c released from mitochondria after OGD exposure. This mitochondrial protein is an early signal of apoptosis that can activate the intrinsic and the extrinsic apoptotic pathways, both of which converge to activate the effector enzyme caspase-3 [24,25,26]. In line with previous evidence [22], the level of cytosolic cytochrome-c was already detectable 2 h after OGD and further increased at 6 h. CSP-1103 significantly prevented cytochrome-c release from 2 h on (Figure 3A–D).

Ibuprofen did not modify the cytochrome release at any time. These results suggest that, conversely to CSP-1103, ibuprofen cannot limit either apoptosis or secondary necrosis in pure neuronal cultures exposed to OGD. The finding is also consistent with previous evidence showing that ibuprofen-induced neuroprotection in brain ischemia is strictly mediated by the reduction of glial reactivity and is not reproduced in pure neuronal cells [27,28].

### 2.4. Effect of CSP-1103 and Ibuprofen, on p38 MAPK, GSK-3β, and NF-κB Activation in Primary Cortical Neurons Exposed to OGD

**Figure 4:**
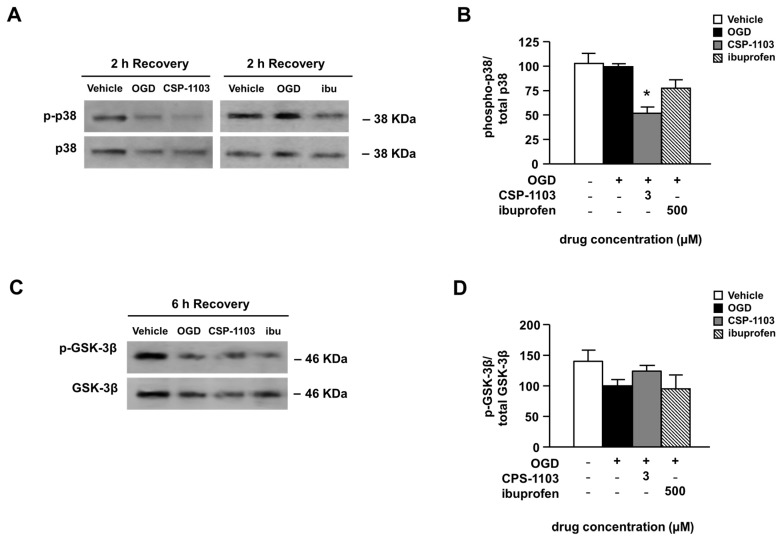
Effect of CSP-1103 and ibuprofen on p38 mitogen-activated protein kinase (MAPK) and glycogen synthase kinase-3β (GSK-3β) in cortical neurons exposed to OGD. (**A**) Representative WB images and (**B**) densitometric analysis of activated p38 form (phospho-p38) and total p38 in the cytosolic extracts of neuronal cells after OGD and 2 h of recovery. CSP-1103, but not ibuprofen, reduced the phospho-p38/p38 ratio. Bars (mean ± SEM) represent the percentage of the phospho-p38/p38 ratio, relative to the OGD value; (**C**) Representative WB images of the inactivated GSK-3β form (phospho-GSK-3β) and the total GSK-3β in the cytosolic extracts of neuronal cells after 6 h of recovery; (**D**) Densitometric analysis represents the ratio between phospho-GSK-3β and total GSK-3β. CSP-1103, but not ibuprofen, reverted the trend to a decrease of the phospho-GSK-3β/GSK-3β ratio after OGD and 6 h of recovery. Bars (mean ± SEM) represent the percentage of the phospho-GSK-3β/GSK-3β ratio, relative to the OGD value. * *p* < 0.05 vs. OGD value. +, presence of OGD; −, absence of OGD or treatment.

Inhibition of p38 mitogen-activated protein kinase (MAPK) activity has been shown to provide neuroprotection in cerebral ischemia [29]. To evaluate the activation state of the p38 MAPK pathway, the ratio between the densitometry value of the activated p38 form (p-p38) and total p38 was calculated. A WB analysis, using antibodies against either the active (p-Thr180/Tyr182) p38 form or total p38, was performed in the cytoplasmic extracts of cells exposed to OGD and 2 h of recovery. In line with previous evidence, no increase in p-p38 was detected in the cortical neurons exposed to OGD [30]. However, 2 h of treatment with CSP-1103 in the post-OGD period strongly reduced the p38 phosphorylation to a level below the basal value (Figure 4A,B). The treatment with ibuprofen did not modify the p38 activation state (Figure 4A,B).

A signaling molecule also involved in the pathogenesis of post-ischemic brain injury is the glycogen synthase kinase-3β (GSK-3β) [31,32]. Since chronic treatment with CSP-1103 was found to reduce the brain level of GSK-3β and concomitantly increase the kinase phosphorylated inactive form in a mouse model of AD [33], we investigated one possible rapid activation of the kinase in neuronal cultures exposed to OGD. The immunoblot analysis of either the inactive (pSer9) GSK-3β or total GSK-3β was performed in cell extracts 6 h after the OGD. The p-GSK-3β/GSK-3β ratio showed only a trend to decrease 6 h after the OGD exposure, consistent with a minor, or just initial, activation of GSK-3β at that time [34]. The trend was reverted by CSP-1103, but not by ibuprofen (Figure 4C,D). No differences were found in the total GSK-3β level both in vehicle and treated neurons exposed to OGD (Figure S2).

**Figure 5:**
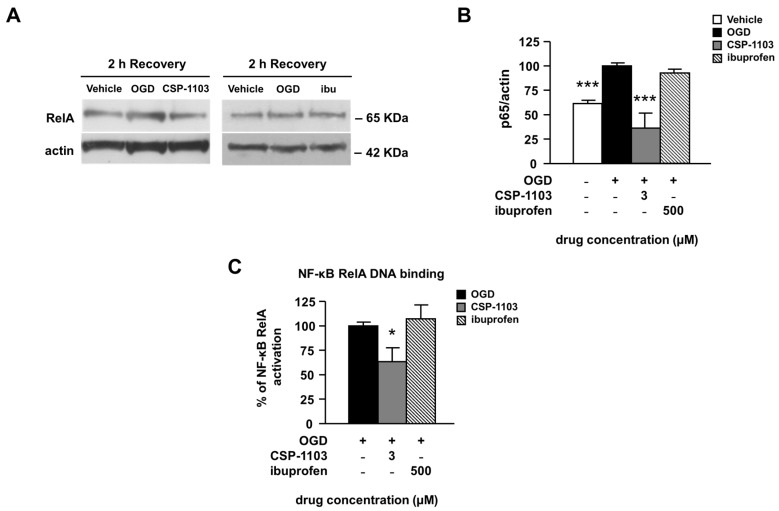
Effect of CSP-1103 and ibuprofen on nuclear levels of RelA in cortical neurons exposed to OGD. (**A**) Representative images and (**B**) densitometric analysis of WB for RelA in nuclear extracts after 2 h recovery. When added after the OGD, only CSP-1103 was able to revert the nuclear increase of RelA. Bars (mean ± SEM) represent the percentage of the RelA/actin ratio, relative to the OGD value; (**C**) ELISA analysis of the DNA binding activity of RelA in nuclear extracts prepared 2 h after the OGD. CSP-1103, but not ibuprofen, reduced the DNA binding activity of RelA. Bars (mean ± SEM) represent the percentage of the DNA binding activity over the basal value, relative to OGD. * *p* < 0.05, *** *p* < 0.001 vs. OGD value. +, presence of OGD; −, absence of OGD or treatment.

Finally, we evaluated the activation of nuclear factor κB (NF-κB), a constitutively expressed transcription factor involved in pro-apoptotic and pro-inflammatory gene expression [35]. We measured the nuclear translocation of NF-κB by WB analysis of the RelA subunit in nuclear extracts from neuronal cells exposed to the vehicle or the drugs in the 2 h post-OGD. CSP-1103, but not ibuprofen, reduced the immunoreactivity of the RelA subunit in nuclear extracts, suggesting a reduced nuclear translocation (Figure 5A,B).

This result was supported by analysis of the DNA binding activity of RelA, measured by DNA-based enzyme-linked immunosorbent assay (ELISA) in nuclear extracts. When compared to the vehicle condition, treatment with CSP-1103, but not ibuprofen, reduced the NF-κB RelA binding activity (Figure 5C).

## 3. Discussion

Since previous studies have shown that CSP-1103 “prevents” the AICD-mediated pro-apoptotic transcription in AD mice [18] and the apoptotic cascade in cultured neurons [20,21], we here explored the neuroprotective potential of CSP-1103 using a post-ischemic paradigm. The effect of CSP-1103 was compared to that of ibuprofen, which has previously been proposed as a neuroprotective agent.

We investigate the capability of CSP-1103 and ibuprofen to interfere with the pro-apoptotic pathways activated in primary cultures of cortical neurons after exposure to OGD. This cell-based model of brain ischemia provides insights into cellular mechanisms of post-ischemic injury and drug activities that have been widely validated by in vivo studies [22,23]. The neuroprotection elicited by CSP-1103, added in the post OGD period, comprised the inhibition of the cytochrome c- and caspase-3 dependent apoptotic cascade, as well as inhibition of necrosis and the p38 and NF-κB signaling pathways.

CSP-1103 reduced the activation of caspase-3 as well as the cytoplasmic release of cytochrome c from mitochondria, which are early markers of apoptotic pathway activation. In addition, cortical neurons exposed to 3 h of OGD and a subsequent 24 h of recovery displayed an increased release of the enzyme LDH, a correlate of late necrotic neuronal death [22,23]. Treatment with CSP-1103 in the recovery period protected neuronal cells from death, as demonstrated by the decrease of LDH in the culture medium. These results are in line with previous studies showing the anti-apoptotic activity of CSP-1103 both in hippocampal neurons exposed to OGD [21] and in the SH-SY5Y cell line treated with Aβ25-35 or TRAIL [20]. Though, while the previous studies investigated the efficacy of CSP-1103 “pretreatment”, the present data provide evidence of the ability of CSP-1103 to limit the apoptotic cascade when added in the post-injury period, a condition important from a potential therapeutic standpoint.

Both p38 MAPK and NF-κB signaling pathways showed a direct involvement in the pathogenesis of brain ischemia [22,29,34,35], also by stimulating the production of pro-inflammatory cytokines [36,37,38]. In cortical neurons, CSP-1103 inhibited the p38 MAPK and NF-κB pathways activation within 2 h after OGD. Conversely, only a minor GSK-3β activation was evident within 6 h after the OGD. At that time point, CSP-1103 was able to reverse the trend without significantly affecting either the inhibitory phosphorylation of GSK-3β or the total GSK-3β content. This result is in line with the unchanged levels of total p-GSK-3β detected in pure cortical neurons exposed for 18 h to CSP-1103 or ibuprofen, in spite of the increased p-GSK-3β/GSK-3β ratio observed in AD mice treated for 6 consecutive months with either drug [33].

Our investigations show that the anti-apoptotic neuroprotective effect of CSP-1103 is not shared by ibuprofen. Ibuprofen showed no direct neuroprotective activity in primary neurons, though when administered in animal models of global ischemia, it was found to decrease neuronal damage, increase cerebral blood flow and ameliorate neurological outcome [28,39,40,41]. In animal models of focal ischemia, ibuprofen reduced infarct size [42,43]. Further studies in mixed cell cultures and brain slices exposed to glutamatergic excitotoxicity or to OGD [27,28,44] established that ibuprofen could limit neuronal cell death, though its activity was strictly dependent on the presence of glial cells [27]. The present study confirms that evidence by showing a lack of interaction with the pro-apoptotic pathway by ibuprofen and a lack of neuroprotection in pure cortical neurons exposed to OGD.

This paper supports multiple lines of evidence showing that the structural modifications designed to eliminate COX inhibitory activity in CSP-1103 produced a compound with unique pharmacological properties. It is reasonable to hypothesize that AICD, a molecular target of CSP-1103, could contribute to the apoptotic pathway and inflammatory process triggered by brain ischemia. While several evidences showed increased APP processing in cerebral regions affected by brain ischemia [45,46], so far the precise role of AICD in stroke pathophysiology and signaling cascades remains almost unexplored. AICD has been identified as a positive regulator of apoptosis because of its transcriptional activation of pro-apoptotic *KAI1*, *p53*, and *GSK-3β* genes [47]. Moreover AICD was found to directly bind and activate cytoplasmic GSK-3β in mouse models overexpressing AICD [48,49]. In the present study, we detected only a non-significant increase in GSK-3β activation at the early time point we considered (6 h after OGD). Although these data seem to exclude the GSK-3β inhibition in the rapid anti-apoptotic effect of CSP-1103, a later involvement of this pathway cannot be excluded. Definitely, whether and how AICD generation affects brain ischemia and is implicated in the neuroprotective activity of CSP-1103 deserves further investigation.

## 4. Materials and Methods

### 4.1. Oxygen-Glucose Deprivation in Nearly Pure Primary Mouse Cortical Neurons

Primary mouse cortical neurons were prepared as previously described [50]. All animal studies were approved by the Animal Research Committees of the University of Brescia and follow the Directive 2010/63/EU of the European Parliament and of the Council of 22 September 2010 on the protection of animals used for scientific purposes. Briefly, C57BL/6J mice were purchased from Charles River, Lecco, Italy. Fifteen-day embryonic mice were harvested with caesarean section from anaesthetized pregnant dams. Cerebral cortices were isolated and dissociated by manual dispersion with a fire-polished Pasteur pipette. The cells were plated in Neurobasal medium supplemented with 2% B27, 0.5 mM l-glutamine, and 50 U/mL penicillin/streptomicin. At 11 days in vitro, neurons were incubated with warm deoxygenated glucose-free balanced salt solution (BSS: KCl 5.36 mM, NaCl 116.35 mM, MgSO_4_ 0.81 mM, and NaH_2_PO_4_ 1.01 mM), transferred to an air-tight chamber, fluxed with an anaerobic gas mixture (95% N_2_ and 5% CO_2_) to remove oxygen, and then incubated at 37 °C for 3 h. At the end of OGD, cortical neurons were allowed to recover in Neurobasal medium containing 0.4% B27 supplement under normoxic conditions and with CSP-1103 (formerly CHF5074) or ibuprofen in 0.2% DMSO or with a vehicle. Cell death was evaluated after 24 h recovery. Protein extraction was performed 2 or 6 h after the OGD. Neuronal cell death by necrosis was evaluated using the CytoTox 96-non-radioactive cytotoxicity assay (Promega, Madison, WI, USA). LDH release was calculated as the amount of LDH released into the culture medium relative to the total releasable LDH, obtained by incubating the cells for 30 min with 1% Triton X-100. Values are expressed as a percentage of LDH released by cells exposed to OGD. The neuronal apoptosis was evaluated by measuring the level of cytochrome c (WB) and cleaved-caspase-3 (immunocytochemistry and WB) at the indicated times.

### 4.2. Immunocytochemistry

After exposure to 3 h of OGD and 24 h of recovery, primary cortical neurons were fixed for 15 min with Immunofix (Bio-Optica, Milan, Italy). Cells were incubated for 15 min with 0.2% Igepal and 0.3% H_2_O_2_ in 0.1 M PBS to inhibit endogenous peroxidases, then blocked for 1 h in 0.1 M phosphate-buffered saline (PBS) containing 3% bovine serum albumin (BSA) and 0.2% Igepal. Neurons were incubated for 2 h at 37 °C with rabbit polyclonal anti-cleaved caspase-3 antibody (1:800, #AF835 R&D, Minneapolis, MN, USA) in 0.1 M PBS containing 3% BSA and 0.2% Igepal. The primary antibody was detected by biotinylated anti-rabbit secondary antibody (1:600, Vector Laboratories, Burlingame, CA, USA) in PBS 0.1 M and 1% BSA, incubated for 1 h in the dark. The signal was revealed by incubation for 45 min in the dark with ABComplex (Vector Laboratories, Burlingame, CA, USA), visualized with 3,3′-Diaminobenzidine (DAB) (Sigma Aldrich, St. Louis, MO, USA) and 1% H_2_O_2_ in 0.1 M PBS. The cells were subsequently counter-stained with hematoxylin, dehydrated in ethanol, and mounted with DPX upon slides. For all procedures except the final DAB reaction, PBS was used as a washing buffer. Quantification of cell apoptosis was performed by counting c-caspase-3 positive cells and hematoxylin stained neurons and data were expressed as percentages of c-casp-3-positive cells to total cell number.

### 4.3. Western Blot Analysis

Analyses of pro-apoptotic proteins in cytosolic extracts were performed as previously described [22,51]. Briefly, cells were resuspended in 100 µL of lysis buffer (KH_2_PO_4_ 1.06 mM, NaCl 155.17 mM, Na_2_HPO_4_·7H_2_O 2.96 mM, KCl 80 mM, sucrose 250 mM, AEBSF 1 mM, aprotinin 10 µg/mL, pepstatin 1 µM, and digitonin 0.1 mg/mL, pH 7.4). They were incubated on ice for 15 min and centrifuged at 15,000× *g* (15 min, 4 °C). The protein lysates (20 µg/sample) were processed for WB analysis using the following primary antibodies; polyclonal anti-caspase-3 antibody (1:500, #9662 Cell Signaling, Danvers, MA, USA), monoclonal anti-cytochrome c antibody (1:300, sc13156 Santa Cruz Biotechnology, Dallas, TX, USA), polyclonal anti-p38 MAPK (1:500, #9212 Cell Signaling, Danvers, MA, USA), monoclonal anti-phospho-p38 MAPK (Thr180/Tyr182) antibody (1:500, #4511 Cell Signaling, Danvers, MA, USA), and polyclonal anti-actin antibody (1:1000, #A5060 Sigma Aldrich, St. Louis, MO, USA).

In order to analyze RelA activation, nuclear extracts were prepared as previously described [50,52]. Briefly, cells were scraped in 400 µL cold Buffer A (10 mM HEPES-KOH pH 7.9 at 4 °C, 1.5 mM MgCl_2_, 10 mM KC1, 0.5 mM dithiothreitol, and 0.2 mM phenylmethanesulfonyl fluoride) and resuspended by flicking the tube. Cells were incubated on ice for 10 min and then centrifuged (10 s). The pellet was processed for high-salt extraction by resuspension in cold Buffer C (20 mM HEPES-KOH pH 7.9, 25% glycerol, 420 mM NaCl, 1.5 mM MgCl_2_, 0.2 mM EDTA, 0.5 mM dithiothreitol, and 0.2 mM PMSF) and incubation on ice for 20 min. Cellular debris was removed by centrifugation for 2 min at 4 °C and the supernatant fraction (containing nuclear proteins) was then stored at −80 °C. For WB analyses, nuclear extracts (25 μg protein/sample) were resolved by 4%–12% SDS/polyacrylamide gel. Immunodetection was performed by incubating the membrane overnight at 4 °C with the primary antibody, polyclonal anti RelA antibody (1:200, sc-372, Santa Cruz Biotechnology, Dallas, TX, USA).

The immunoreaction was revealed by 1 h incubation at 37 °C with secondary antibodies coupled to horseradish peroxidase (HRP) (1:5000, NA934 GE Healthcare, Chicago, IL, USA) and chemiluminescence detection using enhanced chemiluminescence (ECL) western blotting reagents (RPN2132, GE Healthcare, Chicago, IL, USA). Quantification of immunoblots was performed by densitometric scanning of the exposed film using Gel Pro.3 analysis software (MediaCybernetics, Rockville, MD, USA).

### 4.4. DNA-Based ELISA

Binding of mouse RelA to the NF-κB binding consensus sequence was evaluated by the ELISA-based Trans-Am NF-κB kit (Active Motif, Carlsbad, CA, USA). The analysis procedure was performed as recommended by the manufacturer. 30 μg of nuclear extracts were transferred to 96-well plates containing high density immobilized κB oligonucleotides. The active form of the RelA subunit in whole-cell extracts was detected using a specific antibody for the subunit, bound to the target DNA. Incubation with the primary antibody was followed by incubation with the HRP-conjugated secondary antibody. After the addition of developing solutions, the samples were read by spectrophotometry. Data are analyzed by subtracting the absorbance value observed in the presence of nuclear proteins from that obtained in the absence of nuclear proteins.

### 4.5. Statistical Analysis

All results were expressed as mean ± SEM (standard error of the mean). Data were analyzed with one-way analysis of variance (ANOVA), followed by Dunnet’s multiple comparison test. *p* < 0.05 was considered significant.

## 5. Conclusions

In conclusion, this study reveals the ability of CSP-1103 to prevent neuronal death and apoptosis in an in vitro model of post-ischemic brain injury. These data, together with pre-clinical and clinical results showing the capability of the compound to reduce pre-clinical and clinical markers of neuroinflammation [13,14,15,17], suggest that CSP-1103 may have the potential for the treatment of brain ischemia and support the need of detailed studies in animal models of stroke.

## Supplementary Materials

Supplementary materials can be found at www.mdpi.com/1422-0067/18/1/184/s1.

## Abbreviations

TUNEL	terminal deoxynucleotidyl transferase dUTP nick end labeling
NSAID	non-steroidal anti-inflammatory drug
AD	Alzheimer’s disease
Aβ	β-amyloid
sCD40L	soluble CD40 ligand
TNF-α	tumor necrosis factor α
CSF	cerebrospinal fluid
AICD	amyloid precursor protein intracellular domain
TRAIL	tumor necrosis factor related apoptosis inducing ligand
OGD	oxygen-glucose deprivation
LDH	lactate dehydrogenase
c-casp-3	cleaved caspase-3 protein
WB	western blot
MAPK	mitogen-activated protein kinase
GSK-3β	glycogen synthase kinase-3β
NF-κB	nuclear factor kappa B
DAB	3,3′-diaminobenzidine
HRP	horseradish peroxidase

## References

[1] Donnan G.A., Fisher M., Macleod M., Davis S.M. (2008). Stroke. Lancet.

[2] Broughton B.R., Reutens D.C., Sobey C.G. (2009). Apoptotic mechanisms after cerebral ischemia. Stroke.

[3] Benchoua A., Guégan C., Couriaud C., Hosseini H., Sampaïo N., Morin D., Onténiente B. (2001). Specific caspase pathways are activated in the two stages of cerebral infarction. J. Neurosci..

[4] Ferrer I., Friguls B., Dalfó E., Justicia C., Planas A.M. (2003). Caspase-dependent and caspase-independent signalling of apoptosis in the penumbra following middle cerebral artery occlusion in the adult rat. Neuropathol. Appl. Neurobiol..

[5] Duan S.R., Wang J.X., Wang J., Xu R., Zhao J.K., Wang D.S. (2010). Ischemia induces endoplasmic reticulum stress and cell apoptosis in human brain. Neurosci. Lett..

[6] Mitsios N., Gaffney J., Krupinski J., Mathias R., Wang Q., Hayward S., Rubio F., Kumar P., Kumar S., Slevin M. (2007). Expression of signaling molecules associated with apoptosis in human ischemic stroke tissue. Cell Biochem. Biophys..

[7] Rami A., Sims J., Botez G., Winckler J. (2003). Spatial resolution of phospholipid scramblase 1 (plscr1), caspase-3 activation and DNA-fragmentation in the human hippocampus after cerebral ischemia. Neurochem. Int..

[8] Sairanen T., Szepesi R., Karjalainen-Lindsberg M.L., Saksi J., Paetau A., Lindsberg P.J. (2009). Neuronal caspase-3 and parp-1 correlate differentially with apoptosis and necrosis in ischemic human stroke. Acta Neuropathol..

[9] Imbimbo B.P., Del Giudice E., Colavito D., D’Arrigo A., Dalle Carbonare M., Villetti G., Facchinetti F., Volta R., Pietrini V., Baroc M.F. (2007). 1-(3′,4′-dichloro-2-fluoro[1,1′-biphenyl]-4-yl)-cyclopropanecarboxylic acid (CHF5074), a novel gamma-secretase modulator, reduces brain β-amyloid pathology in a transgenic mouse model of Alzheimer’s disease without causing peripheral toxicity. J. Pharmacol. Exp. Ther..

[10] Balducci C., Mehdawy B., Mare L., Giuliani A., Lorenzini L., Sivilia S., Giardino L., Calzà L., Lanzillotta A., Sarnico I. (2011). The γ-secretase modulator CHF5074 restores memory and hippocampal synaptic plasticity in plaque-free TG2576 mice. J. Alzheimers Dis..

[11] Imbimbo B.P., Hutter-Paier B., Villetti G., Facchinetti F., Cenacchi V., Volta R., Lanzillotta A., Pizzi M., Windisch M. (2009). Chf5074, a novel gamma-secretase modulator, attenuates brain β-amyloid pathology and learning deficit in a mouse model of Alzheimer’s disease. Br. J. Pharmacol..

[12] Sivilia S., Lorenzini L., Giuliani A., Gusciglio M., Fernandez M., Baldassarro V.A., Mangano C., Ferraro L., Pietrini V., Baroc M.F. (2013). Multi-target action of the novel anti-Alzheimer compound chf5074: In vivo study of long term treatment in TG2576 mice. BMC Neurosci..

[13] Imbimbo B.P., Frigerio E., Breda M., Fiorentini F., Fernandez M., Sivilia S., Giardino L., Calzà L., Norris D., Casula D. (2013). Pharmacokinetics and pharmacodynamics of CHF5074 after short-term administration in healthy subjects. Alzheimer Dis. Assoc. Disord..

[14] Porrini V., Lanzillotta A., Branca C., Benarese M., Parrella E., Lorenzini L., Calzà L., Flaibani R., Spano P.F., Imbimbo B.P. (2014). CHF5074 (CSP-1103) induces microglia alternative activation in plaque-free Tg2576 mice and primary glial cultures exposed to β-amyloid. Neuroscience.

[15] Ross J., Sharma S., Winston J., Nunez M., Bottini G., Franceschi M., Scarpini E., Frigerio E., Fiorentini F., Fernandez M. (2013). CHF5074 reduces biomarkers of neuroinflammation in patients with mild cognitive impairment: A 12-week, double-blind, placebo-controlled study. Curr Alzheimer Res.

[16] Town T., Nikolic V., Tan J. (2005). The microglial “Activation” Continuum: From innate to adaptive responses. J. Neuroinflamm..

[17] Imbimbo B.P., Fernandez M., Giardino L., Calzà L., Chain D., Margolin R. Relationship between cerebrospinal fluid (CSF) biomarkers and cognitive performance of patients with mild cognitive impairment (MCI) after long-term treatment with chf5074. Proceedings of the 17th Alzheimer’s Association International Conference, Alzheimer's & Dementia 2014.

[18] Branca C., Sarnico I., Ruotolo R., Lanzillotta A., Viscomi A.R., Benarese M., Porrini V., Lorenzini L., Calzà L., Imbimbo B.P. (2014). Pharmacological targeting of the β-amyloid precursor protein intracellular domain. Sci. Rep..

[19] Pardossi-Piquard R., Checler F. (2012). The physiology of the β-amyloid precursor protein intracellular domain aicd. J. Neurochem..

[20] Ronsisvalle N., di Benedetto G., Parenti C., Amoroso S., Bernardini R., Cantarella G. (2014). CHF5074 protects sh-sy5y human neuronal-like cells from amyloidβ 25–35 and tumor necrosis factor related apoptosis inducing ligand toxicity in vitro. Curr. Alzheimer Res..

[21] Mango D., Barbato G., Piccirilli S., Panico M.B., Feligioni M., Schepisi C., Graziani M., Porrini V., Benarese M., Lanzillotta A. (2014). Electrophysiological and metabolic effects of CHF5074 in the hippocampus: Protection against in vitro ischemia. Pharmacol. Res..

[22] Sarnico I., Lanzillotta A., Boroni F., Benarese M., Alghisi M., Schwaninger M., Inta I., Battistin L., Spano P., Pizzi M. (2009). NF-κB p50/rela and c-rel-containing dimers: Opposite regulators of neuron vulnerability to ischaemia. J. Neurochem..

[23] Valerio A., Dossena M., Bertolotti P., Boroni F., Sarnico I., Faraco G., Chiarugi A., Frontini A., Giordano A., Liou H.C. (2009). Leptin is induced in the ischemic cerebral cortex and exerts neuroprotection through NF-κB/C-Rel-dependent transcription. Stroke.

[24] Adams J.M., Cory S. (2002). Apoptosomes: Engines for caspase activation. Curr. Opin. Cell Biol..

[25] Ghavami S., Hashemi M., Ande S.R., Yeganeh B., Xiao W., Eshraghi M., Bus C.J., Kadkhoda K., Wiechec E., Halayko A.J. (2009). Apoptosis and cancer: Mutations within caspase genes. J. Med. Genet..

[26] Fuchs Y., Steller H. (2011). Programmed cell death in animal development and disease. Cell.

[27] Iwata Y., Nicole O., Zurakowski D., Okamura T., Jonas R.A. (2010). Ibuprofen for neuroprotection after cerebral ischemia. J. Thorac. Cardiovasc. Surg..

[28] Park E.M., Cho B.P., Volpe B.T., Cruz M.O., Joh T.H., Cho S. (2005). Ibuprofen protects ischemia-induced neuronal injury via up-regulating interleukin-1 receptor antagonist expression. Neuroscience.

[29] Sugino T., Nozaki K., Takagi Y., Hattori I., Hashimoto N., Moriguchi T., Nishida E. (2000). Activation of mitogen-activated protein kinases after transient forebrain ischemia in gerbil hippocampus. J. Neurosci..

[30] Bhuiyan M.I., Jung S.Y., Kim H.J., Lee Y.S., Jin C. (2011). Major role of the PI3K/Akt pathway in ischemic tolerance induced by sublethal oxygen-glucose deprivation in cortical neurons in vitro. Arch. Pharm. Res..

[31] Zhang X., Wang C., Li Y., Dong L., Cui L., Wang L., Liu Z., Qiao H., Zhu C., Xing Y. (2012). Neuroprotection of early and short-time applying berberine in the acute phase of cerebral ischemia: Up-regulated PAKT, PGSK and PCREB, down-regulated NF-κB expression, ameliorated BBB permeability. Brain Res..

[32] Kelly S., Zhao H., Hua Sun G., Cheng D., Qiao Y., Luo J., Martin K., Steinberg G.K., Harrison S.D., Yenari M.A. (2004). Glycogen synthase kinase 3β inhibitor Chir025 reduces neuronal death resulting from oxygen-glucose deprivation, glutamate excitotoxicity, and cerebral ischemia. Exp. Neurol..

[33] Lanzillotta A., Sarnico I., Benarese M., Branca C., Baiguera C., Hutter-Paier B., Windisch M., Spano P., Imbimbo B.P., Pizzi M. (2011). The γ-secretase modulator CHF5074 reduces the accumulation of native hyperphosphorylated tau in a transgenic mouse model of Alzheimer’s disease. J. Mol. Neurosci..

[34] Takahashi-Yanaga F. (2013). Activator or inhibitor? GSK-3 as a new drug target.. Biochem. Pharmacol..

[35] Lanzillotta A., Porrini V., Bellucci A., Benarese M., Branca C., Parrella E., Spano P.F., Pizzi M. (2015). NF-κB in innate neuroprotection and age-related neurodegenerative diseases. Front. Neurol..

[36] Barone F.C., Irving E.A., Ray A.M., Lee J.C., Kassis S., Kumar S., Badger A.M., Legos J.J., Erhardt J.A., Ohlstein E.H. (2001). Inhibition of p38 mitogen-activated protein kinase provides neuroprotection in cerebral focal ischemia. Med. Res. Rev..

[37] Guo R.B., Wang G.F., Zhao A.P., Gu J., Sun X.L., Hu G. (2012). Paeoniflorin protects against ischemia-induced brain damages in rats via inhibiting MAPKS/NF-κB-mediated inflammatory responses. PLoS ONE.

[38] Harari O.A., Liao J.K. (2010). Nf-κb and innate immunity in ischemic stroke. Ann. N. Y. Acad. Sci..

[39] Grice S.C., Chappell E.T., Prough D.S., Whitley J.M., Su M., Watkins W.D. (1987). Ibuprofen improves cerebral blood flow after global cerebral ischemia in dogs. Stroke.

[40] Kuhn J.E., Steimle C.N., Zelenock G.B., d’Alecy L.G. (1986). Ibuprofen improves survival and neurologic outcome after resuscitation from cardiac arrest. Resuscitation.

[41] Patel P.M., Drummond J.C., Sano T., Cole D.J., Kalkman C.J., Yaksh T.L. (1993). Effect of ibuprofen on regional eicosanoid production and neuronal injury after forebrain ischemia in rats. Brain Res..

[42] Antezana D.F., Clatterbuck R.E., Alkayed N.J., Murphy S.J., Anderson L.G., Frazier J., Hurn P.D., Traystman R.J., Tamargo R.J. (2003). High-dose ibuprofen for reduction of striatal infarcts during middle cerebral artery occlusion in rats. J. Neurosurg..

[43] Cole D.J., Patel P.M., Reynolds L., Drummond J.C., Marcantonio S. (1993). Temporary focal cerebral ischemia in spontaneously hypertensive rats: The effect of ibuprofen on infarct volume. J. Pharmacol. Exp. Ther..

[44] López-Villodres J.A., De La Cruz J.P., Muñoz-Marin J., Guerrero A., Reyes J.J., González-Correa J.A. (2012). Cytoprotective effect of nonsteroidal antiinflammatory drugs in rat brain slices subjected to reoxygenation after oxygen-glucose deprivation. Eur. J. Pharm. Sci..

[45] Salminen A., Kauppinen A., Kaarniranta K. (2016). Hypoxia/ischemia activate processing of amyloid precursor protein: Impact of vascular dysfunction in the pathogenesis of Alzheimer’s disease. J. Neurochem..

[46] Ułamek-Kozioł M., Pluta R., Bogucka-Kocka A., Januszewski S., Kocki J., Czuczwar S.J. (2016). Brain ischemia with alzheimer phenotype dysregulates alzheimer’s disease-related proteins. Pharmacol. Rep..

[47] Chang K.A., Suh Y.H. (2010). Possible roles of amyloid intracellular domain of amyloid precursor protein. BMB Rep..

[48] Trazzi S., Fuchs C., de Franceschi M., Mitrugno V.M., Bartesaghi R., Ciani E. (2014). App-dependent alteration of GSK3β activity impairs neurogenesis in the ts65dn mouse model of down syndrome. Neurobiol. Dis..

[49] Zhou F., Gong K., Song B., Ma T., van Laar T., Gong Y., Zhang L. (2012). The app intracellular domain (aicd) inhibits wnt signalling and promotes neurite outgrowth. Biochim. Biophys. Acta.

[50] Lanzillotta A., Pignataro G., Branca C., Cuomo O., Sarnico I., Benarese M., Annunziato L., Spano P., Pizzi M. (2013). Targeted acetylation of NF-κB/Rela and histones by epigenetic drugs reduces post-ischemic brain injury in mice with an extended therapeutic window. Neurobiol. Dis..

[51] Movsesyan V.A., Stoica B.A., Faden A.I. (2004). Mglur5 activation reduces β-amyloid-induced cell death in primary neuronal cultures and attenuates translocation of cytochrome c and apoptosis-inducing factor. J. Neurochem..

[52] Andrews N.C., Faller D.V. (1991). A rapid micropreparation technique for extraction of DNA-binding proteins from limiting numbers of mammalian cells. Nucleic. Acids Res..

