# Supplementary material for: Neuroprotective and Anti-Apoptotic Effects of CSP-1103 in Primary Cortical Neurons Exposed to Oxygen and Glucose Deprivation
Source: Int J Mol Sci. 2017 Jan 18;18(1):184. doi: 10.3390/ijms18010184 (PMC5297816; doi:10.3390/ijms18010184)
Supplement: Supplementary file 1 [file ijms-18-00184-s001.pdf]

Supplementary Materials: Neuroprotective and Anti-Apoptotic Effects of CSP-1103 in Primary Cortical Neurons Exposed to Oxygen and Glucose Deprivation

Vanessa Porrini, Ilenia Sarnico, Marina Benarese, Caterina Branca, Mariana Mota, Annamaria Lanzillotta, Arianna Bellucci, Edoardo Parrella, Lara Faggi, Pierfranco Spano, Bruno Pietro Imbimbo and Marina Pizzi

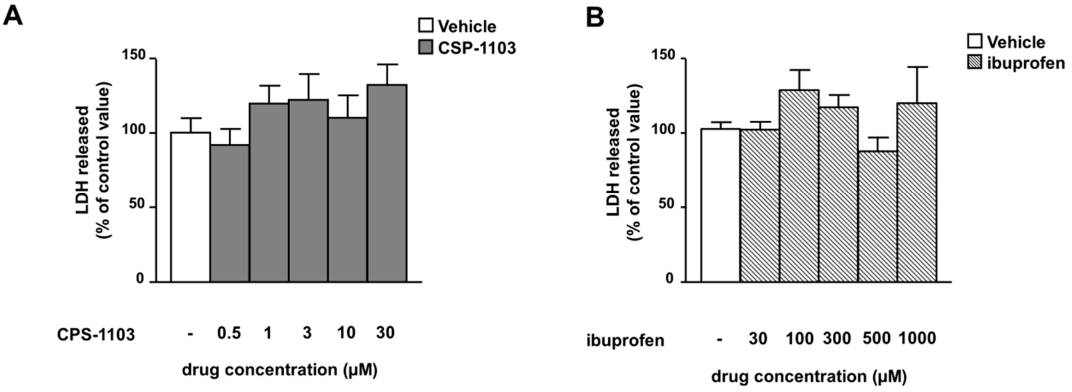

Figure S1. Absence of toxic effect of CSP-1103 and ibuprofen in naïve culture of cortical neurons. (**A**) Cortical neurons were exposed to different doses of CSP-1103 for 24 h and neuronal death was assessed by the lactate dehydrogenase (LDH) assay. None of the different doses of CSP-1103 increased the basal level of LDH release from neurons; (**B**) Lack of toxic effect of ibuprofen in control culture of cortical neurons. Different doses of ibuprofen were added for 24 h and the neuronal death was measured by the LDH assay. Values are expressed as a percentage of LDH released by naive cells. −, absence of treatment.

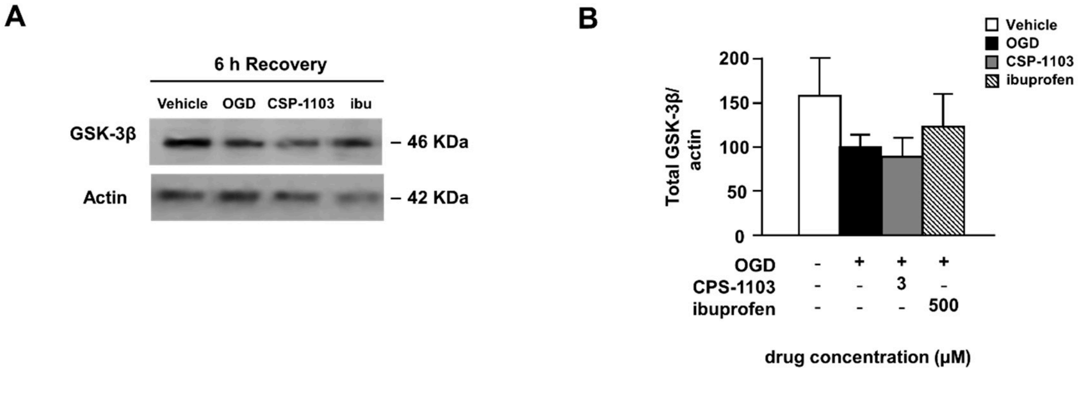

Figure S2. Effect of CSP-1103 and ibuprofen on glycogen synthase kinase-3β (GSK-3β) in cortical neurons exposed to oxygen glucose deprivation (OGD). (**A**) Representative western blot images of GSK-3β in the cytosolic extracts of neuronal cells after 6 h of recovery; (**B**) Densitometric analysis represents the ratio between GSK-3β and actin. Neither CSP-1103 nor ibuprofen affected the GSK-3β expression. Bars (mean ± SEM) represent the percentage of the GSK-3β/actin ratio, relative to the OGD value. +, presence of OGD; −, absence of OGD or treatment.
